# Cytoplasmic crystal inclusions in lymphocytes of chronic lymphocytic leukemia

**DOI:** 10.1002/jha2.118

**Published:** 2020-10-28

**Authors:** Òscar Villuendas Vázquez, Maria José Herranz Martí, Pável Olivera

**Affiliations:** ^1^ Clinical Analysis Department Sant Pau i Santa Tecla Hospital Tarragona Spain; ^2^ Department of Hematology Sant Pau i Santa Tecla Hospital Tarragona Spain; ^3^ Division of Clinical Hematology Banc de Sang i Teixits Barcelona Spain; ^4^ Sant Pau Biomedical Research Institute (IIB Sant Pau) Barcelona Spain

A 50‐year‐old male was attended in 2007 because of an absolute lymphocytosis of 10 × 10^9^/L. Surface markers by flow cytometry were positive for cluster of differentiation (CD) 20, CD23, CD19, and CD5, and negative for CD10, CD103, CD38, and zeta‐chain‐associated protein kinase 70 (ZAP70), with kappa light chain restriction.

**FIGURE 1:**
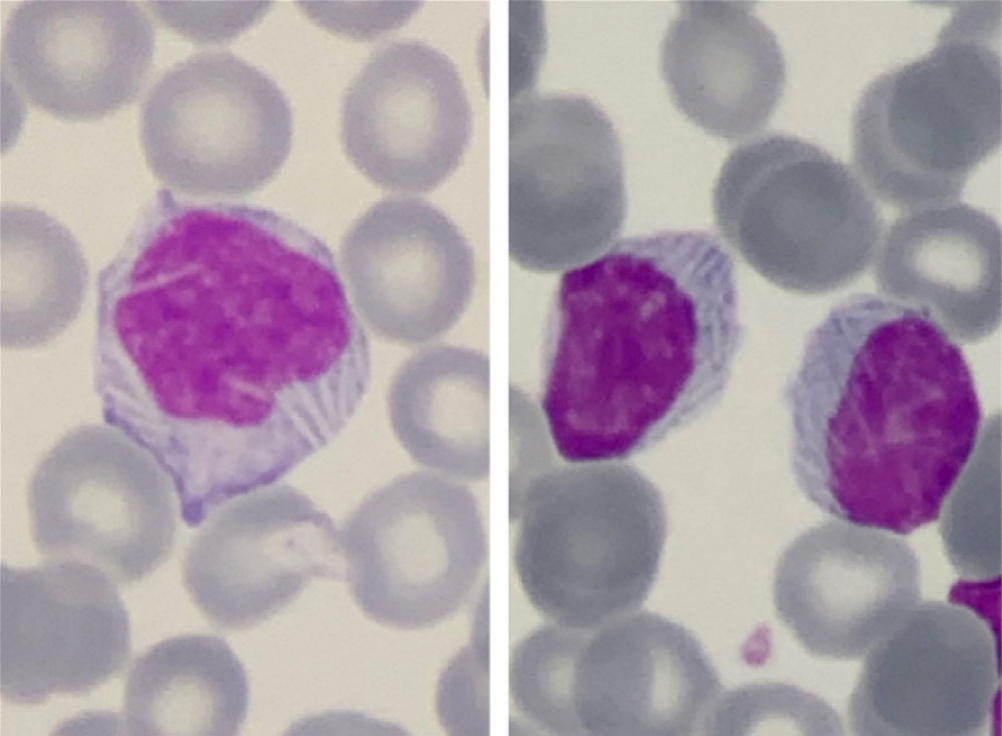
May‐Grünwald‐Giemsa stain; 100x magnification

**FIGURE 2:**
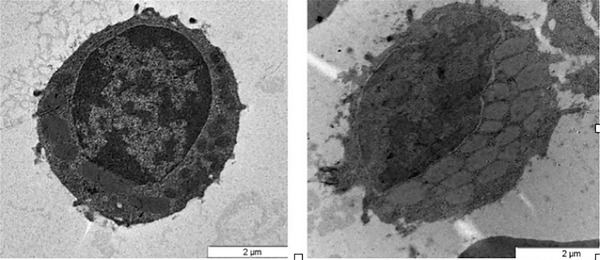
Ultrastructure of two lymphocytes showing cytoplasmic inclusions in different sections (TEM, original magnification 25 000x)

Karyotype was normal, while fluorescence in situ hybridization (FISH) revealed a deletion of chromosome 13 (del13q14) in 15% of the nuclei. The patient was asymptomatic and no lymph nodes were palpable. May‐Grünwald‐Giemsa stained peripheral blood smear examination showed mild‐sized abnormal lymphocytes with irregular nuclei and moderate cytoplasm. Several intracytoplasmic pale inclusions, crystal‐like shaped, were seen in 78% of lymphocytes (Figure 1). Transmission electron microscopy (TEM) images were collected using a JEOL 1011 Transmission Electron Microscope operating at 80 kV and showed several electron‐dense rectangular inclusions in the cytoplasm (Figure 2). The patient was diagnosed with chronic lymphocytic leukemia (CLL) Binet stage A, with no markers of unfavorable prognosis.

During the follow up, the number of lymphocytes has been increasing slowly.

Currently, 13 years after diagnosis, the patient remains off treatment and has evolved to Binet stage B because of mild lymphadenopathy. Current white blood cells are 57 × 10^9^/L, with 80% lymphocytes, and the same cytoplasmic inclusions in 75% of them.

Few cases of cytoplasmic inclusions in CLL have been described in the literature [1, 2, 3], and most believed to be of proteic nature of immunoglobulins. It is not clear, if any, the meaning of this feature.

Herein in our case, we conclude that this morphologic variant does not seem to confer worse prognosis “per se” in CLL.
